# The Development of the Psychological Monitors’ Burnout Scale in College

**DOI:** 10.3390/bs16071190

**Published:** 2026-07-15

**Authors:** Qisheng Zhan, Fanglin Song, Xinyi Liu, Yonghui Li, Shanji Li, Jia Li, Zheng Wang, Jingjing Liu, Min Zhou, Shiyong Liu, Hui Liu

**Affiliations:** 1 Institute of Psychology, Tianjin University, Tianjin 300072, China; sfl2023@tju.edu.cn (F.S.); lxy_2026@tju.edu.cn (X.L.); 2 School of Education, Tianjin University, Tianjin 300350, China; 3 Psychological Counseling Center, East China University of Science and Technology, Shanghai 200237, China; liyonghui@ecust.edu.cn; 4 Mental Health Education and Counseling Center, Yanbian University, Yanji 133000, China; sjli1@ybu.edu.cn; 5 Mental Health Education Center, Northwest University, Xi’an 710069, China; lijia@nwu.edu.cn; 6 Mental Health Education Center, North China Electric Power University, Beijing 102206, China; wangzheng@ncepu.edu.cn; 7 Mental Health Education and Service Center, Xinjiang Normal University, Urumqi 830017, China; liujj@xjnu.edu.cn; 8 Mental Health Education Center, Yangzhou University, Yangzhou 225009, China; zhoumin@yzu.edu.cn; 9 Mental Health Education Center, Fuyang Normal University, Fuyang 236037, China; liushiyong@fynu.edu.cn; 10 Student Counseling and Mental Health Center, Peking University, Beijing 100871, China

**Keywords:** psychological monitors, burnout, development of scale, validity, reliability

## Abstract

The aim of this study was to develop the Psychological Monitor’s Burnout Scale in College (PMBSC) and test its validity and reliability. Based on literature analysis, open-ended questionnaire surveys and interviews, the construct of PMBSC was defined and the preliminary scale was compiled in this study. In total, 530 psychological monitors were selected for item analysis and exploratory factor analysis; another 859 were selected for confirmatory factor analysis, split-half reliability and internal consistency reliability tests, of which 172 were retested after 5 weeks. The Competency Questionnaire for Psychological Monitors in College (CQPMC) and Chinese Perceived Stress Scale (CPSS) were used to test the criterion validity. The PMBSC contained 20 items and was composed of three factors that accounted for 64.71% of the variance. The confirmatory factor analysis showed that the three-factor structure model fitted well (χ^2^/df = 5.53, RMSEA = 0.073, CFI = 0.938, NFI = 0.926, RFI = 0.916, IFI = 0.938, TLI = 0.930, GFI = 0.899). The criterion validity test showed that the total scores and factor scores of PMBSC were negatively correlated with the total scores of CQPMC (r = −0.54–−0.65, *p* < 0.01), while they were positively correlated with the total scores of CPSS (r = 0.39–0.48, *p* < 0.01). The Cronbach’s α coefficients of the total scale and its three factors ranged from 0.88 to 0.95, the split-half reliability ranged from 0.88 to 0.94, and the test–retest reliability ranged from 0.72 to 0.85. The Psychological Monitor’s Burnout Scale in College has good validity and reliability. The PMBSC provides targeted measurement tools for college psychological monitors and verifies the adaptability of Maslach’s three-dimensional burnout model among student peer helpers theoretically. Practically, the scale supports regular burnout screening and precise mental health intervention for psychological monitor teams in universities.

## 1. Introduction

### 1.1. Psychological Monitor System and Burnout Phenomenon

In 2004, to effectively address the increasingly prominent mental health issues among college students, Tianjin University explored and established a psychological monitor system with Chinese characteristics through practical implementation. This class-based peer support system aligns closely with the interpersonal functioning and mental well-being dimensions of the WHO International Classification of Functioning, Disability and Health (ICF) biopsychosocial framework. Unlike voluntary peer counseling programs widely adopted in Western universities that offer reactive mental health support upon student requests, Chinese psychological monitors are embedded in every class, undertaking regular proactive mental health screening, universal mental health literacy promotion, and early psychological crisis identification, forming a whole-campus preventive mental health network with broader coverage and earlier intervention. This system achieves student self-education, self-service, and self-growth by selecting class-level student leaders to dedicate themselves to addressing students’ mental health needs and undertaking mental health education initiatives at both the university and departmental levels (Ma & Ouyang, 2020). Psychological monitors serve as role models for maintaining their own psychological well-being, advocates for disseminating basic mental health knowledge, identifiers of students facing psychological crises, and supporters for safeguarding their peers’ mental health (Zhan et al., 2022). Therefore, they play a pivotal role in preventing, detecting, identifying, and addressing psychological crises among college students (Zhan & Yao, 2023). Given the remarkable effectiveness of the psychological monitor system, universities nationwide have followed suit and incorporated it into their mental health service systems. According to the 2024 national survey data on psychological monitors’ work in universities, among the 2617 participating institutions, the total number of psychological monitors reached 1,131,597, averaging 3 psychological monitors per 100 college students (Zhan & Liu, 2024). Meanwhile, as the number of psychological monitors continues to grow, a trend of burnout has emerged within the group. This is manifested in the following ways: (1) Emotional resource depletion. Tian’s study of 420 university psychological monitors found that first-year undergraduate psychological monitors scored highest on the emotional support dimension of interpersonal communication ability, while third-year undergraduate psychological monitors scored lowest (Tian, 2015), indicating that psychological monitors’ emotional resources gradually diminish over time. Yue Peina also discovered that factors such as heavy workload and high professional requirements led some psychological monitors to experience anxiety, depression, and other negative emotions (Yue, 2021). (2) Negative cognition. Wang conducted open-ended interviews with eight psychological monitors and found that they generally lacked enthusiasm and expectations for their work, held negative evaluations of their work, and believed their work had limited efficacy (D. Wang, 2021). (3) Weakened work motivation. Liao et al.’s study of 854 psychological monitors from six universities in Fujian Province found that some psychological monitors experienced a “career plateau” phenomenon after serving for two to three years, with this group showing significantly decreased scores in work motivation and work attitude dimensions compared to novice psychological monitors (Liao et al., 2017). The above phenomena are typical characteristics of burnout, indicating that some psychological monitors’ work performance has declined over time.

### 1.2. Burnout Theories and Existing Measurement Tools

Burnout was first proposed by the American psychologist Freudenberger in 1974, who defined it as a state of physical, emotional, and psychological exhaustion resulting from prolonged emotional involvement (Freudenberger, 1974). Building upon extensive clinical experience, Maslach expanded and refined this concept, defining burnout as a long-term psychological response arising from individuals’ inability to effectively cope with continuous work-related stressors. Burnout encompasses emotional exhaustion, depersonalization, and reduced personal accomplishment (Maslach & Jackson, 1981; Maslach et al., 2001). Maslach also argued that burnout predominantly occurs in helping professions, such as physicians, nurses, and psychological counselors. These professionals require frequent interaction with recipients and substantial emotional investment, and this sustained emotional labor easily leads to resource depletion, causing them to experience a sense of burnout. While Maslach’s approach captures the multifaceted nature of burnout, the alternative JD-R model, advanced by Demerouti and Schaufeli, takes a more parsimonious stance by condensing the syndrome into two core dimensions. Specifically, it defines burnout as a state of exhaustion and disengagement, which results from the chronic depletion of energy caused by high job demands and the lack of sufficient resources to offset these demands (Demerouti et al., 2001, 2003; Schaufeli & Bakker, 2004). These two theoretical frameworks represent the dominant mainstream interpretations of burnout, yet scholars have not reached a unified consensus regarding the dimensional structure and core connotation of this construct. Adding to this complexity, the World Health Organization’s International Classification of Diseases (ICD-11), revised in 2019, classifies burnout as an “occupational phenomenon” resulting from “chronic workplace stress that has not been successfully managed,” characterized by three dimensions: energy depletion, mental distance from one’s job, and reduced professional efficacy (World Health Organization, 2019). More critically, a body of scholars argues that burnout may not qualify as a unique, separate construct entirely. Bianchi and colleagues have argued that the MBI “fails to measure the construct it is purported to measure” and that the problems affecting the MBI “question the very idea of burnout” (Bianchi et al., 2024). De Witte and Schaufeli refute Bianchi and Schonfeld’s radical skepticism toward burnout, confirming burnout as a work-related distinct construct separable from depression and arguing the widespread “burnout pandemic” claim is exaggerated by mixing mild self-reported burnout symptoms with rare clinical burnout disorders (De Witte & Schaufeli, 2025). Given this ongoing controversy, the present study adopts a clearly defined theoretical stance. We align with Maslach’s multidimensional framework, which conceptualizes burnout as comprising emotional exhaustion, depersonalization, and reduced personal accomplishment. This framework was chosen because the psychological monitor role in Chinese colleges is characterized by public service, peer support, and substantial emotional labor—precisely the type of helping profession for which the Maslach model was originally designed.

Many scholars from psychology and management have developed multiple relatively mature burnout measurement instruments based on their respective research perspectives and structural theories. Among these, the Maslach Burnout Inventory (MBI) series developed by Maslach is currently the world’s most widely applied and highly recognized burnout measurement tool. Maslach conceptualized burnout as comprising three dimensions: emotional exhaustion, depersonalization, and reduced personal accomplishment. Based on this framework, three versions of the scale were developed: MBI-HSS (Human Services Survey), MBI-ES (Educators Survey), and MBI-GS (General Survey) (Maslach et al., 1997). Additionally, there are burnout measurement tools developed based on single-dimensional and two-dimensional frameworks. The Burnout Measure (BM) includes only the “exhaustion” dimension (Pines & Aronson, 1988), though its reliability and validity lack sufficient empirical research. The Oldenburg Burnout Inventory (OLBI), developed based on the two dimensions of “exhaustion” and “disengagement”, with each dimension employing both positively and negatively worded items (Demerouti et al., 2003), overcoming certain limitations of the MBI to some extent.

Burnout scales are mostly revised or developed based on the MBI in China. C. Li et al. were among the earliest Chinese scholars to introduce and validate the MBI locally. After administering it to Chinese enterprise employees, they found that the MBI-GS possessed good structural validity (C. Li & Shi, 2003). They removed one item from the cynicism dimension of the original scale, ultimately retaining 15 items. Subsequently, Li’s team revised the MBI-HSS (C. Li et al., 2003) and MBI-ES (C. Li & Wang, 2009). They argued that the MBI has cultural adaptation issues, and that existing Chinese revised versions of the MBI maintained consistency with the original scale structure by sacrificing certain questionnaire items. Therefore, they developed the CMBI based on Chinese cultural context (Y. Li & Wu, 2005). Following this, the focus of burnout measurement research in China gradually shifted from translating and revising foreign scales to independently developing more applicable measurement tools that incorporate local cultural characteristics and occupational specificity, such as the Job Burnout Questionnaire in primary and secondary school teachers (Xu et al., 2004), the Job Burnout Scale for Competition Sports Coaches (Yin & Xue, 2009), and the Job Burnout Scale—General Survey (Guo & Xu, 2017).

### 1.3. Limitations of Prior Scales and Study Rationale

Psychological monitors not only face students’ counseling needs but also assist in handling emergent incidents and urgent situations, all of which consume their emotional resources. In addition, given the nature of their work, psychological monitors often need to provide services after class or during leisure time, further exacerbating the imbalance between resource consumption and recovery, thus leading to burnout. Burnout is closely associated with critical occupational outcomes among psychological monitors, including diminished job satisfaction (Cheng et al., 2022; Lushin et al., 2023; Rutherford et al., 2023), reduced organizational commitment (Tuvilla & Potane, 2023; Chen & Liu, 2021), and increased turnover intention. Second, psychological monitors’ negative emotions may further spread, affecting other students’ psychological states and ultimately adversely impacting the entire class’s mental health level. Therefore, timely detection and regulation of university psychological monitors’ burnout levels is particularly important, yet currently there is no measurement tool specifically designed for psychological monitors.

Existing burnout scales and their Chinese revised versions are developed for full-time paid practitioners such as teachers and professional counselors, so they cannot capture the unique burnout experience of college psychological monitors. Psychological monitors hold a dual identity as ordinary undergraduates and unpaid peer volunteers, lacking systematic professional training, steady salaries and standardized institutional management. They suffer from intertwined academic burden and volunteer service demands, including routine emotional communication, mental health publicity and crisis screening—stressors that are absent in formal occupational groups.

Simple superficial revision of existing scales cannot fundamentally solve this mismatch. Minor wording tweaks only alter occupational descriptions and fail to reflect the unique psychological conflicts brought by the student–volunteer dual identity. Unlike full-time employees with complete organizational support, psychological monitors’ burnout stems from the tension between coursework and unpaid public service, a contextual feature that cannot be fully reflected by existing scale frameworks. Critically, the psychological monitor system is a distinctive Chinese campus mental health prevention model, whose service objects, working modes and incentive mechanisms differ greatly from Western peer counselors and domestic professional mental health staff. Only an indigenous scale developed from open questionnaires and interview data that record their authentic burnout feelings can accurately reflect their burnout structural features.

In summary, burnout symptoms have emerged among some psychological monitors. Considering the complexity of psychological monitors’ roles and the special nature of their work, existing burnout measurement tools are difficult to apply directly to this population. The absence of specialized measurement tools has become the primary reason why research on university psychological monitors’ burnout cannot be conducted in depth. Therefore, this study proposes to develop a reliable burnout measurement instrument for college psychological monitors based on Maslach’s burnout structural theory, grounded in literature review, and employing methods including open-ended questionnaire surveys, semi-structured interviews, and factor analysis. This instrument will provide an entry point for subsequent interventions targeting psychological monitors’ burnout levels. The overall scale development and validation framework of this study draws on internationally recognized general psychometric guidelines for construct definition, item screening and validity assessment proposed by Lambert and Newman (2023), Zickar (2020), and Stefana et al. (2025), with detailed operational steps adjusted to fit the unique peer volunteer background of college psychological monitors.

## 2. Materials and Methods

### 2.1. Participants

Sample 1: Predictive Questionnaire Survey. Using convenience sampling, five universities were selected from five provinces and cities: Anhui, Hebei, Guangdong, Beijing, and Tianjin. Faculty members responsible for psychological monitors at each institution were tasked with distributing questionnaire links to psychological monitors through online platforms. Psychological monitors voluntarily clicked the links to participate in the survey. A total of 678 psychological monitors completed the online questionnaire, with 530 valid responses collected. Among them, 308 were first-year students, 130 were second-year students, 82 were third-year students, and 10 were fourth-year students. Their ages ranged from 18 to 28, with an average age of 20 ± 1 years; 463 students (87.4%) were elected as psychological monitors through competitive campaigns, while 67 students (12.6%) were appointed to the role. The average tenure for psychological monitors was 14 months.

Sample 2: Formal Questionnaire Survey. Using convenience sampling, eight universities were selected from eight provinces and cities: Jilin, Xinjiang, Jiangsu, Sichuan, Shaanxi, Hebei, Henan, and Tianjin. Teachers responsible for psychological monitors’ work at each university or college were commissioned to distribute questionnaire links to psychological monitors through online platforms, and psychological monitors voluntarily clicked the links to participate in the survey. A total of 1262 psychological monitors completed the online questionnaire, with 859 valid questionnaires collected. There were 284 male and 575 female participants. Among them, 224 were first-year students, 294 were second-year students, 193 were third-year students, 110 were fourth-year students, and 38 were postgraduates. Their ages ranged from 17 to 28 years, with a mean age of 20 ± 2 years; 747 students (87.0%) served as psychological monitors through election, while 112 students (13.0%) were appointed. The average tenure as psychological monitors was 13 months.

Additionally, 172 participants were conveniently selected for test–retest reliability assessment with a 5-week interval, including 57 males and 115 females. The sample consists of 90 first-year students, 46 second-year students, 22 third-year students, 6 fourth-year students, and 8 postgraduates. Their ages ranged from 17 to 27 years, with a mean age of 19 ± 2 years; 151 students (87.8%) served as psychological monitors through election, while 21 students (12.2%) were appointed. The average tenure as psychological monitors was 8 months.

### 2.2. Procedure of Development of the Questionnaire

#### 2.2.1. Open-Ended Questionnaire and Interviews

Using convenience sampling, four universities were selected from four provinces and cities: Fujian, Anhui, Tianjin, and Zhejiang. Teachers responsible for psychological monitors affairs at each institution were commissioned to distribute open-ended questionnaire links to psychological monitors through online platforms. Psychological monitors voluntarily clicked the links to participate in the survey. A total of 588 valid questionnaires were collected, including 155 male students and 433 female students. Among them, 314 were first-year students, 164 were second-year students, 85 were third-year students, and 25 were fourth-year students; 535 students (91.0%) were elected to serve as psychological monitors, while 53 (9.0%) were appointed. The average tenure as a psychological monitor was 11 months. The open-ended questionnaire included the following items: (1) What is your overall experience during your tenure as a psychological monitor? (2) Compared to when you first took the role of psychological monitors, how has your work state changed? (3) During your tenure, how severe was your work burnout (1 = low, 9 = high)? (4) What signs might indicate burnout among psychological monitors? (5) What do you think causes burnout among psychological monitors? (6) What impacts might burnout among psychological monitors potentially have?

To gain deeper insights into the work burnout among college psychological monitors, semi-structured interviews were conducted with a sample selected from those who participated in an open-ended questionnaire survey. Sampling ceased once the principle of information saturation was met, meaning no new categories emerged. A total of 10 individuals were interviewed, 4 males and 6 females; 7 interviews were conducted online, and 3 were conducted in person. The average tenure as a psychological monitor was 16 months. The interview questions included: (1) What has been your overall experience serving as a psychological monitors? (2) Have you experienced negative burnout during your tenure? If so, what were the specific manifestations? (3) (For second-year, third-year and fourth-year psychological monitors) Compared to your first year, how has your work status changed as a psychological monitor? (4) What do you believe are the causes of burnout among psychological monitors? (5) How do you view the work you have done in the past?

Based on grounded theory, open-ended questionnaire data and interview transcripts were coded and analyzed using Nvivo 11.0 software. Through multiple rounds of induction and synthesis, the analysis yielded 73 free codes, 11 associated codes, and 4 core codes. Associative codes included severe negative emotions (68, referring to frequency, same as below), mental and physical exhaustion (58), lack of initiative (51), superficial work performance (91), passive detachment (92), low sense of belonging (66), lack of pleasure (14), low sense of efficacy (48), and knowledge depletion (57). Among these, negative emotions and being stretched too thin were categorized as “emotional exhaustion”; lack of initiative, perfunctory work, and avoidance behaviors were grouped as “depersonalization”; low sense of presence, lack of pleasure, and low efficacy were classified as “reduced personal accomplishment”; and knowledge depletion was identified as “knowledge depletion”. Based on a review of the relevant literature and the above coding results, a preliminary structural model of the psychological monitor’s burnout scale in college was developed. This model comprises four dimensions: emotional exhaustion, depersonalization, reduced personal accomplishment, and knowledge depletion.

#### 2.2.2. Item Generation Phase and Expert Review

Data from open-ended questionnaires and semi-structured interviews are coded. Four core qualitative themes together with three-level sub-codes under each theme are obtained. Separate item drafting frameworks are established for each theme, and all preliminary items are compiled by referring to the original contextual information of each coded segment. Combined with the preliminary structural model of burnout among college psychological monitors, a total of 48 items are generated. Being drawn from similar dimensions in the Chinese-revised MBI-GS (C. Li & Shi, 2003) on the basis of the MBI-HSS (Maslach, 1996), Primary and Secondary School Teacher Burnout Questionnaire (X. Wu et al., 2016), and Competitive Sports Coach Burnout Scale (Yin & Xue, 2009), six additional items were developed, resulting in a final set of 54 items. One expert and nine psychology master’s degree holders conducted item-by-item semantic discussions and analyses, revising or deleting items with ambiguous meanings, unclear or unreasonable expressions, or interpretive ambiguities. The item “I face too much pressure” was deleted. The item “Balancing my studies and duties as psychological monitors leaves me feeling stretched too thin” was revised to “I feel stretched too thin when balancing my studies and duties as psychological monitors.” The item “Exposure to too much negative emotion from classmates leaves me feeling drained” was revised to “I feel drained due to exposure to too much negative emotion from classmates.” There are 53 items in the final preliminary version of the Psychological Monitor’s Burnout Scale in College. The questionnaire employs a 4-point Likert scale (1 = Strongly Disagree, 2 = Disagree, 3 = Agree, 4 = Strongly Agree).

### 2.3. Criterion Questionnaire

#### 2.3.1. Competency Questionnaire for Psychological Monitors in College (CQPMC)

The Competency Questionnaire for Psychological Monitors in College developed by Zhan et al. (2021). This 35-item questionnaire encompasses five dimensions: role identification, communication and interaction, self-mental state, professional ethics, and negative traits. It utilizes a 4-point scoring scale ranging from 1 (strongly disagree) to 4 (strongly agree), with higher scores indicating greater competency levels among psychological monitors. In this study, the Cronbach α coefficient for the questionnaire was 0.95, with each factor’s Cronbach α coefficients ranging from 0.60 to 0.92.

#### 2.3.2. Chinese Perceived Stress Scale (CPSS)

The Chinese version of the Perceived Stress Scale revised by Yang and Huang was adopted (Yang & Huang, 2003). This 14-item scale comprises two dimensions: tension and loss of control. It employs a 5-point scoring method ranging from 1 (never) to 5 (always), with higher scores indicating greater perceived stress levels. In this study, the Cronbach α coefficient for the scale was 0.85, with each factor’s Cronbach α coefficients ranging from 0.78 to 0.81.

### 2.4. Data Processing

Using SPSS 27.0, item analysis and exploratory factor analysis were conducted on Sample 1. The internal consistency reliability, split-half reliability, test–retest reliability (Intraclass Correlation Coefficient, ICC), and criterion validity analysis were performed on Sample 2. Confirmatory factor analysis was conducted on Sample 2 using Amos 27.0.

### 2.5. Ethical Considerations

This study complied with the Declaration of Helsinki and relevant national ethical regulations. All participants provided electronic informed consent prior to surveys and interviews, with full anonymity guaranteed and the right to withdraw freely. No identifiable personal information was collected, and the research posed no physical or mental risks to participants. Per national rules for low-risk anonymous research, ethical review exemption applies, and all data were securely stored for academic use only.

## 3. Results

### 3.1. Item Analysis of the Initial Questionnaire

A total of 53 preliminary items retained after expert semantic review in Section 2.2.2 were used for subsequent item analysis. This study employed the critical ratio method (CR), correlation method, and the homogeneity reliability method to select items (M. Wu, 2010). One item was deleted due to a critical ratio (CR) was less than 3 or statistically insignificant score differences between high and low groups (*p* > 0.05), leaving 52 items. Among the remaining 52 items, 3 items with reduced total score correlations were removed, leaving 49 items. Finally, the remaining 49 items underwent homogeneity reliability screening. Four items were deleted if their adjusted total item correlations were <0.4 or if removing them increased the total scale’s Cronbach α coefficient. Ultimately, 45 items were selected for exploratory factor analysis. These 45 candidate items then entered the exploratory factor analysis stage, where items were further eliminated to form the final 20-item scale.

### 3.2. Validity Analysis

#### 3.2.1. Construct Validity

The 45 candidate items retained after item analysis in Section 3.1 were submitted to exploratory factor analysis. The KMO value for the 45-item scale was 0.96, and Bartlett’s sphericity test yielded a value of 15,453.44 (df = 990, *p* < 0.001), indicating suitability for factor analysis. Principal component analysis and maximum variance extraction were employed to obtain the factor loading matrix. Item selection criteria were: (1) factor loadings less than 0.45; (2) fewer than 3 items on the same factor; (3) simultaneous loadings greater than 0.40 on two factors; and (4) items within the same factor whose meanings differed from other items. After repeated exploration of the factor structure, 20 items were ultimately retained. The three factors were named: reduced personal accomplishment, emotional exhaustion, and dehumanization, explaining 64.71% of the total variance. The variance contribution rates for each factor and the standardized factor loadings for each item are shown in Table 1 and Table 2.

Confirmatory factor analysis of the formal questionnaire: The three-factor structure obtained from exploratory factor analysis was validated, and the scale’s fit indices met psychometric requirements (χ^2^/df = 5.53, RMSEA = 0.073, CFI = 0.938, NFI = 0.926, RFI = 0.916, IFI = 0.938, TLI = 0.930, GFI = 0.899). The standardized loadings of each item on its respective factor ranged from 0.69 to 0.86.

#### 3.2.2. Criterion Validity Analysis of the Formal Questionnaire

Conservation of Resources theory posits that individuals possess various resources that are crucial for maintaining physical, mental, and life balance. When individuals face stress, resources are continuously depleted, and if resource depletion continues without replenishment, individuals will experience burnout (Hobfoll, 1989). Competence is an extremely important resource possessed by individuals. Therefore, this study selected the Competency Questionnaire for Psychological Monitors in College (CQPMC) and Chinese Perceived Stress Scale (CPSS) of college psychological monitors as criteria for PMBSC to examine its criterion validity.

The total PMBSC score and scores for each factor were negatively correlated with the total CQPMC score (120.9 ± 14.4), while they were positively correlated with the total CPSS score (32.62 ± 8.0) (Table 3).

### 3.3. Reliability Analysis of the Formal Questionnaire

This study conducted reliability analysis of the PMBSC formal questionnaire using internal consistency reliability, split-half reliability, and test–retest reliability. The internal consistency reliability (Cronbach α coefficient) of the total scale was 0.95. The Cronbach α coefficients for the three factors—reduced personal accomplishment, emotional exhaustion, and depersonalization—were 0.92, 0.93, and 0.88, respectively. The split-half reliability of the total scale was 0.91, while the split-half reliability coefficients for the three factors were 0.92, 0.94, and 0.88, respectively. The test–retest reliability of the total scale was 0.85, with coefficients for the three factors being 0.81, 0.83, and 0.72, respectively. All indices greater than 0.7 indicate that the PMBSC demonstrates good reliability.

## 4. Discussion

### 4.1. The Structural Dimensional Characteristics of Burnout Among Psychological Monitors

Based on the analysis of previous literature, open-ended questionnaire surveys, and semi-structured interview results, this study preliminarily established a four-factor structural model of burnout among university psychological monitors and developed the initial Psychological Monitor Burnout Scale for College students (PMBSC). Exploratory factor analysis further refined three factors of burnout among university psychological monitors: “reduced personal accomplishment, emotional exhaustion and depersonalization”. The factor “reduced personal accomplishment” refers to psychological monitors’ lack of sense of achievement, competence, and self-efficacy in their work, negating the meaning and value of their work. The factor “emotional exhaustion” refers to psychological monitors feeling exhausted and emotionally depleted due to prolonged exposure to work-related stress, being prone to negative emotions such as tension, fear, and frustration in their work. The factor “depersonalization” refers to psychological monitors not viewing their classmates as individuals with emotions and needs, displaying negative, indifferent, neglectful, and avoidant attitudes toward them, and losing enthusiasm and interest in their work. It is noteworthy that the results of exploratory factor analysis merged some items from the initial model’s “reduced personal accomplishment” and “knowledge depletion” factors into a new “reduced personal accomplishment” factor. The possible reasons for this are as follows: (1) Knowledge depletion describes the draining sense that the mental health knowledge mastered by psychological monitors cannot meet the diverse and complex psychological demands of their peers. Both knowledge depletion and reduced personal accomplishment center on insufficient self-efficacy in solving psychological service problems, resulting in close conceptual overlap and high correlation between the two latent dimensions according to the EFA results of the present study. From Maslach’s theoretical framework, this provides one of the most plausible interpretations: knowledge depletion may serve as a potential antecedent or concrete manifestation of reduced personal accomplishment (Maslach & Jackson, 1981). (2) Previous literature has identified an independent knowledge depletion factor primarily among teachers and coaches, groups that rely on comprehensive professional knowledge to address complicated work situations (Cheng et al., 2022; W. Li & Zhu, 2019; F. Wang & Xu, 2004). Unlike full-time professional counselors, psychological monitors only deal with relatively simple daily emotional troubles and bear no responsibility for intervening in severe mental disorders such as depression. In line with the original interview transcripts of this research, participants’ descriptions of inadequate knowledge reserves always appeared alongside feelings of low counseling achievement. Knowledge depletion may not form a stable independent dimension within the burnout structural model for this volunteer student sample.

The structural model of university psychological monitor burnout constructed in this study is consistent with the MBI series scales (Maslach et al., 1997), indicating that Maslach’s three-dimensional burnout structural model is equally applicable to the population of university psychological monitors. Compared to the original MBI scale and its revised version, the PMBSC items are formulated to better align with the work context of psychological monitors. Furthermore, variance contribution analysis reveals that the “reduced personal accomplishment” factor in the PMBSC serves as the core of the entire scale, explaining the greatest variance among all factors, whereas the “emotional exhaustion” factor holds this position in the MBI. Possible reasons for this difference include the following: (1) The special nature of college psychological monitors’ roles. In this study, over 85% of psychological monitors were elected through competitive selection. This group often possesses more proactive traits and higher levels of psychological capital (J. Wang et al., 2023), making them potentially more sensitive to positive aspects such as self-efficacy, personal achievement, and growth. In contrast, traditional MBI series scales sampled workplace professionals from various industries who face diverse emotional stressors, not all of whom chose their professions out of passion, with varying levels of psychological capital and weaker moderating effects, making emotional exhaustion the core factor of burnout. (2) Institutional support for psychological monitors’ work within universities. The 2024 national survey on psychological monitors’ work in universities showed that among the 2617 participating universities, the vast majority had relatively comprehensive training, assessment, and incentive measures for psychological monitors; 64.2% of universities had standardized operational mechanisms of “department counselor–class psychological monitor” (Zhan & Liu, 2024). Wang and colleagues’ research also found that psychological monitors received extremely high support from psychological monitors’ organizations (J. Wang et al., 2023). Support from universities and psychological monitors’ organizations can effectively buffer the impact of emotional stress on psychological monitors, and communication and comparison with psychological monitors’ peers may prompt them to focus more on their work efficacy. In contrast, workplace professionals may receive weaker organizational support. (3) The special feedback mechanism for college psychological monitors’ work outcomes. Psychological monitors’ work has public service characteristics; the 2024 national survey on psychological monitors’ work in universities showed that 66.1% of universities’ primary incentive measure for psychological monitors was awarding them the title of “Outstanding Psychological Monitor” (Zhan & Liu, 2024). However, existing research indicates that the overall competency of psychological monitors remains at a moderately high level (J. Wang et al., 2023; Zhan et al., 2021). This suggests that the rewards for their work are predominantly non-material, with motivation largely intrinsic. Consequently, the sense of accomplishment derived from helping others becomes the core factor influencing the level of work burnout among this group.

### 4.2. Psychometric Properties of PMBSC

The confirmatory factor analysis results indicate that all fit indices of the psychological monitors work burnout structural model fall within the acceptable range in psychometrics, demonstrating good model fit. This suggests that PMBSC possesses strong construct validity. This study used the Competency Questionnaire for Psychological Monitors in College (CQPMC) and Chinese Perceived Stress Scale (CPSS) as criterion measures for validity analysis of PMBSC. The results showed that PMBSC total scores and all dimensions were negatively correlated with CQPMC total scores and positively correlated with CPSS total scores, consistent with previous research (Liu et al., 2024; Zeng et al., 2016), indicating that this scale possesses good criterion-related validity. Additionally, the PMBSC demonstrated internal consistency reliability, split-half reliability, and test–retest reliability that meet psychometric standards, indicating the scale’s reliability. Notably, among the PMBSC’s three factors, the dehumanization factor exhibited relatively lower test–retest reliability. Whether this suggests the factor is more sensitive to temporal or historical events requires further investigation in subsequent research.

### 4.3. Limitations of the Present Study and Future Research Directions

This research has several limitations: (1) It relies solely on cross-sectional self-report data from Chinese samples, which may lead to common method bias and cannot infer causal relationships. Longitudinal tracking and multi-source evaluation can clarify causal links and reduce self-report bias. (2) Participants were recruited via teacher-assisted convenience sampling, which may introduce selection bias and restrict sample representativeness; future research should employ more rigorous sampling strategies to further verify the generalizability of the scale. Furthermore, the psychological monitor system is a campus mental health mechanism with distinctive Chinese educational characteristics, so the present findings cannot be directly extended to peer counselor groups under different cultural and educational systems. Future research should adopt more rigorous sampling strategies to further test the cross-sample generalizability of this scale. (3) Principal component analysis (PCA) was adopted as the initial dimension-reduction technique in exploratory factor analysis. Different from common factor extraction methods designed for latent construct exploration, PCA treats all item variance as shared variance, which brings certain methodological limitations when exploring the latent burnout structure of psychological monitors. (4) Regarding content validity, this study only adopted expert semantic review to evaluate item appropriateness without quantitative content validity index calculation or cognitive interviews. This relatively preliminary content validation procedure may limit the rigorousness of item screening. (5) The depersonalization dimension exhibits relatively low test–retest reliability. Scholars should further explore the temporal and contextual factors affecting depersonalization.

## 5. Conclusions

This study developed a 20-item three-dimensional PMBSC and verified its acceptable psychometric properties grounded in Maslach’s burnout theory, offering a specialized burnout measurement tool for Chinese college student psychological monitors. Theoretically, this work provides preliminary empirical evidence for the adaptability of Maslach’s classic three-dimensional burnout model among student peer helpers. Practically, the PMBSC can be utilized for routine burnout screening and targeted mental health intervention within university psychological monitor teams.

## Figures and Tables

**Table 1:** Variance contribution rates of each factor.

Factors	Items	Variance Contribution Rates	Cumulative Variance Contribution Rate
Reduced personal accomplishment	8	24.80%	24.80%
Emotional exhaustion	7	23.73%	48.53%
Depersonalization	5	16.18%	64.71%

**Table 2:** Standardized factor loadings of each item.

Reduced Personal Accomplishment		Emotional Exhaustion		Depersonalization	
Item	Load	Item	Load	Item	Load
37	0.81	29	0.80	22	0.76
35	0.79	46	0.78	44	0.74
10	0.72	38	0.76	14	0.69
11	0.72	4	0.75	25	0.64
8	0.70	20	0.75	45	0.61
30	0.67	52	0.73		
40	0.66	16	0.68		
31	0.65				

**Table 3:** Correlation between PMBSC and criterion measures.

	M ± SD	CQPMC	CPSS
Total score	27.0 ± 8.1	−0.65 **	0.48 **
Reduced personal accomplishment	11.9 ± 4.4	−0.60 **	0.44 **
Emotional exhaustion	9.2 ± 3.1	−0.54 **	0.41 **
Depersonalization	6.0 ± 1.7	−0.58 **	0.39 **

PMBSC, Psychological Monitor’s Burnout Scale in College; CQPMC, Competency Questionnaire for Psychological Monitors in College; CPSS, Chinese Perceived Stress Scale; ** *p* < 0.01.

## Data Availability

The datasets generated and/or analyzed during the current study are not publicly available due to the need to protect the privacy of college student participants, but are available from the corresponding author on reasonable request.

## Abbreviations

The following abbreviations are used in this manuscript:

PMBSC	Psychological Monitors’ Burnout Scale
CQPMC	Competency Questionnaire for Psychological Monitors in College
CPSS	Chinese Perceived Stress Scale
MBI	Maslach Burnout Inventory
